# Lipid accumulation in response to nitrogen limitation and variation of temperature in *Nannochloropsis salina*

**DOI:** 10.1186/s40529-015-0085-7

**Published:** 2015-04-08

**Authors:** Eman M Fakhry, Dahlia M El Maghraby

**Affiliations:** Department of Botany and Microbiology, Faculty of Science, Alexandria University, Alexandria, 21511 Egypt

**Keywords:** Lipids and triglycerides accumulation, Nitrogen depletion, Temperature, Nannochloropsis salina

## Abstract

**Background:**

This batch study deals with the relation between lipid as well as triglyceride contents in *Nannochloropsis salina* and variation in culture conditions such as nitrogen concentration and temperature.

**Results:**

The tested parameters caused reduction in growth expressed as cell count, optical density and dry weight, as well strongly involved in lipids and triglycerides accumulation and significantly affected the lipid productivity. At the beginning of the work, the concentration of nitrogen in the medium was reduced to three quarter, half and quarter of the original f2 medium while the temperature kept constant. After that, the optimal nitrogen concentration (quarter of the original media) giving high lipid yield was tested with different temperature degrees from 15 to 35°C with five degree intervals. Although the growth was insignificantly influenced, a considerable increase in lipid and triglyceride (56.1 and 15.1% of dry weight respectively) was observed when the concentration of nitrogen in the medium was reduced to the quarter. Moreover, 59.3% lipid and 17.1% triglyceride on the basis of dry weight were obtained by the combination of 25% nitrogen concentration and 30°C. Simple regressions recommended that the interaction effect of nitrogen limitation and temperature on lipid and triglyceride accumulation was not as fundamental as for nitrogen limitation stress.

**Conclusion:**

The degree of nitrogen availability in the combination of temperature effect has been identified as the critical determinant for the maximal production of lipid in *N. salina*. Nevertheless, major advances in this field can be considered by studying more stresses techniques and genetic strategies.

**Electronic supplementary material:**

The online version of this article (doi:10.1186/s40529-015-0085-7) contains supplementary material, which is available to authorized users.

## Background

Lipid-producing microalgae have the potential to permit lipid accumulation without demanding for arable land. Under favorable growth conditions, algal biomasses are successfully produced with relatively low lipid contents. Optimization of biomass and lipid in microalgae has been studied in batch, closed and open outdoor systems (Zou and Richmond [2000]; Sandnes et al. [2005]; Grobbelaar [2007]). In those culture systems, the environmental parameters and nutrient supply were found to be vital factors for growth improvement and induction of lipid in microalgae. Accordingly high lipid productivity which is a main requirement for commercial production of microalgal oil-derived biodiesel was achieved (Sharma et al. [2012]). However, increase in lipid productivity (mass of lipids per unit of area per unit of time) can be achieved by either enhancement the rate of biomass accumulation, or increase the biomass with high lipid percent on the basis of dry weight. To attain this goal, different approaches including nutrient starvation, bioprocess optimization and genetic engineering can be used (Hu et al. [2008]). However, the maximum yield will be obtained by the algal response to the surroundings.

Under environmental stress conditions, many algae modify their lipid biosynthetic pathways to produce and accumulate neutral lipids, mainly in the form of triacylglycerol. However, the biosynthesis pathway of triacylglycerol may play active role in the stress response. On the other hand, it offers carbon and energy storage function in the cell that allows microalgae to tolerate unfavorable environmental conditions (Hu et al. [2008]). In general, algal biomass and triglycerides compete for photosynthetic assimilate and a renovating of physiological pathways is required to stimulate lipid biosynthesis (Sharma et al. [2012]). The need to improve lipid production can potentially be addressed by survey microalgae with adequate lipid content. Microalgae are hopeful lipid-producers as they are adapted to grow over a wide range of environmental conditions with short doubling times (Mata et al. [2010]), higher growth rate and photosynthetic efficiencies than conventional crops (Chisti [2007]; Wu et al. [2013]). Furthermore, they possess various and infrequent patterns of cellular lipids and have the ability to alter lipid metabolism in response to environmental changes by several factors, of which nutrients (Chen et al. [2011]; Feng et al. [2011]; Mairet et al. [2011]), temperature (Li et al. [2011]; Fuentes-Grünewald et al. [2012]) and irradiance (Hu et al. [2008]).

Numerous studies highlight the key role of nitrogen and temperature in algal lipid accumulation and the conditions applied in these studies differ extensively. Nitrogen- deficient conditions can affect the microalgal growth and change the metabolic pathway to the accumulation of storage lipids (Lombardi and Wangersky [1991]). Actually, the increase in lipid content may be species and strain specific (El-Baky et al. [2004]; Pal et al. [2011]; Olofsson et al. [2014]). *Dunaliella*, *Chlorella* and *Nannochloropsis* species are known to respond to nitrogen starvation by increasing lipid production (Lombardi and Wangersky [1995]; Guevara et al. [2005]; Converti et al. [2009]; Dong et al. [2013]). Also, it is evident that nitrogen is a limiting factor for the growth of many living species (Hansen et al. [2000]; Li et al. [2010]; Park et al. [2012]). Temperature is one of the principal factors in culture conditions that affect the growth and lipid production in microalgae (Tzovenik et al. [2003]; Roleda et al. [2013], Rukminasari [2013]). The response of microalgal lipid content to high and low growth temperatures differs from species to species (Renaud et al. [2002]; Wu et al. [2013]).

Genus *Nannochloropsis* is a microalga belonging to Eustigmatophyceae. The genus is broadly well appreciated in aquaculture (Roncarati et al. [2004]; Bentley et al. [2008]) due to its comparatively high growth rate, resistance to mixing and contamination together with high nutritional values and high lipid content (Rodolfi et al. [2003]; Olofsson et al. [2012]).

Current study aimed to obtain experimental data that clarify how *N. salina* will respond to stress conditions for maximum production of lipids as biodiesel feedstock and as component for valuable foodstuff and health products. Algal biomass and corresponding total lipid and triglyceride contents were estimated under nitrogen depletion in growth medium with variation in the temperature degrees during cultivation.

## Methods

### Organism and growth condition

*N. salina* was obtained from the Culture Collection of the Med Algae laboratory, Faculty of Science, Alexandria University, Egypt. All the glassware and media were always sterilized prior to cultivation. The cultures were grown in 1 L Erlenmeyer flasks with 600 mL f/2 medium (Guillard [1975]) using the atmospheric CO_2_ as carbon source and kept under controlled environmental conditions of approximately light intensity (150 μmol m^−2^ s^−1^), light/dark cycle (12:12 h) and temperature (25 ± 2°C). The cultures were shaken twice daily to avoid sticking. This was referred to as control culture.

### Experimental design

To investigate the effects of nitrogen deficiency on biomass and lipid yield, *N. salina* was batch cultured autotrophically and axenically in N-deficient conditions using the original source of nitrogen in f/2 medium (sodium nitrate). N-deficiency was achieved by cultivation of the microalga in conical flasks containing 1 L. of culture medium with normal nitrogen concentration and three additional cultivations were run under N-deficient conditions (25, 50 and 75% of the original levels used in the control medium). Subsequently, re-cultivation of cells in further experiment was provided to evaluate the effect of temperature under the chosen N concentration. The temperature values tested were from 15 to 35°C with five degree intervals. The media were buffered with sodium bicarbonate and HCl to pH 7.5. Each treatment consisted of triplicate flasks and continuous aeration was provided.

### Growth measurements

The cell density of *N. salina* in culture system was estimated daily by counting under light microscope using the haemocytometer counting chamber. The biomass of the cultures was determined for every 24 h by measuring the optical density at wavelength of 680 nm (Huang et al. [2002]) via Perkin-Elmer spectrum RXIFT-IR System. Each sample was measured twice and the mean value was calculated. To estimate the dry weight, microalgal biomass was harvested by centrifugation at 2000 g for 15 min. The cell pellets were washed twice with distilled water. The collected pellets were oven dried at 60°C till constant weight. The weights of the dry mass were evaluated in relation to the relative biomass of cultures.

### Extraction of total cellular lipids and triglycerides

Lipid content was estimated at zero time and after 12 days (exponential phase) of cultivation. The algal lipid was extracted following the protocol of Bligh and Dyer ([1959]). The cells were harvested by centrifugation at 2000 g for 15 min. The pellet was subjected to wet weight estimation and then dried in oven at 60°C till constant weight. Algal sample was extracted with chloroform: methanol mixture (2:1 v/v) and kept for 24 hours at 25°C. The mixture was vigorously agitated in vortex for few minutes. The homogenate was centrifuged at 4000 rpm for 15 min. The lower layer was separated and the procedure was again repeated with the pellet. The phase containing dissolved lipids were transferred into a separatory funnel and shaken for 5 min. The lipid fractions were separated in a clean pre-weighed vial (first wt) and the solvent was evaporated using rotary evaporator. The weight of the vial was again recorded (second wt). Lipid content was calculated by subtracting first wt from second wt. Triglycerides were extracted using n-hexane, purified by separation on thin layer chromatography and quantified by comparing to a standard curve generated from known amounts of TAG standard. The weight of both lipid content and triglyceride was determined and calculated in correlation to the dry algal biomass. Lipid productivity was calculated as follows:

$$P_{\mathrm{Lipid}}\left(g\;L^{-1}d^{-1}\right)=\frac{C_{f}\times DCW_{f}-C_{i}\times DCW_{i}}{T}$$

Where: P_Lipid_: lipid productivity, C_f_: final lipid content after 12 days of cultivation, DCW_f_: final biomass of the microalgae in the lipid producing phase after 12 days of cultivation, C_i_: initial lipid content at zero time, DCW_i_: initial biomass in the lipid producing phase at zero time, T: the cultivation time.

### Statistical analysis

All experiments were repeated three times independently, and data were recorded as the mean. Statistical analyses were performed using simple linear regressions (R) (version 2.12.0, R Foundation for Statistical Computing, Vienna, Austria) to estimate the relationship between the dependent variables (total lipid and triglyceride contents) and the independent variables (nitrogen concentrations (%) and temperatures). The effects of the treatments were tested by one-way analysis of variance (ANOVA). Means were compared between the treatments using the LSD (least significant difference) test at the 0.05 probability level.

## Results

### Effect of nitrogen limitation

Growth measurements of *N. salina* grown under normal condition and different nitrogen concentrations were reported in Table 1. The micro-alga shows maximum cell count (×10^6^/ml), optical density (OD_680_) and dry weight (g L^−1^) on the twelfth day. The highest cell number obtained was under control condition (9.9 × 10^6^/ml culture) with 0.77 optical density, 0.61 g L^−1^ dry weight and 0.228 g L^−1^ day^−1^ lipid productivity. Media with 75% and 50% nitrogen concentration yielded cell number of 9.1 and 8.2 × 10^6^/ml culture giving 0.57 and 0.53 g L^−1^ dry weight and 0.237 and 0.298 g L^−1^ day^−1^ lipid productivity respectively. The lowest cell number, optical density and dry weight obtained were in medium with 25% nitrogen concentration (7.9 × 10^6^/ml culture, 0.63 and 0.48 g L^−1^ respectively) with highest lipid productivity of 0.370 g L^−1^ day^−1^.

**Table 1:** **Growth measurements and lipid productivity of** ***Nannochloropsis salina*** **operated at different nitrogen concentrations and temperatures**

	Cell count	Optical density	Dry weight of cells	Lipid productivity
	(×10^6^/ml)	(OD_680_)	(g L^−1^)	(g L^−1^ day^−1^)
Control	9.93 ± 0.25	0.77 ± 0.04	0.61 ± 0.03	0.23 ± 0.0
75	9.07 ± 0.45^a*^	0.71 ± 0.03^a*^	0.57 ± 0.06	0.24 ± 0.0^a*^
50	8.23 ± 0.45^a*b*^	0.65 ± 0.02^a*b*^	0.53 ± 0.05^a*^	0.30 ± 0.0^a*b*^
25	7.86 ± 0.45^a*b*^	0.63 ± 0.02^a*b*^	0.48 ± 0.02^a*b*^	0.37 ± 0.0^a*b*c*^
Temperature°C				
15	6.87 ± 0.55	0.53 ± 0.03	0.42 ± 0.05	0.31 ± 0.0
20	7.57 ± 0.35^a^	0.59 ± 0.02^a^	0.45 ± 0.04	0.35 ± 0.01^a^
25	8.23 ± 0.25^ab^	0.66 ± 0.01^ab^	0.50 ± 0.04^a^	0.37 ± 0.01^ab^
30	8.67 ± 0.06^ab^	0.71 ± 0.03^abc^	0.53 ± 0.02^ab^	0.43 ± 0.0^abc^
35	8.57 ± 0.25^ab^	0.72 ± 0.02^abc^	0.51 ± 0.02^a^	0.41 ± 0.01^abcd^

Mean ± SE (n = 3) followed by different letters indicate significant difference at P ≤ 0.05, according to one-way analysis of variance (ANOVA) and the LSD (least significant difference) test (a*: significant with control at ≤0.05, b*: significant with 75% N at ≤0.05, c*: significant with 50% N at ≤0.05, a: significant with 15°C at ≤0.05, b: significant with 20°C at ≤0.05, c: significant with 25°C at ≤0.05, d: significant with 30°C at ≤0.05).

**Figure 1:**
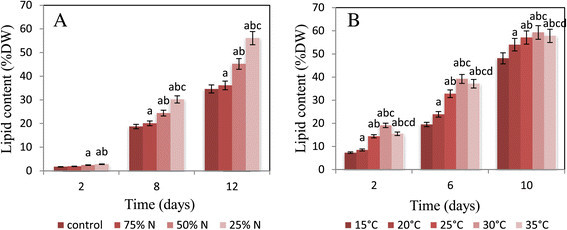
**Cellular lipid content of** ***Nannochloropsis salina*** **.** The lipid contents were monitored **(A)** after 2, 8 and 12 days of incubation under optimal conditions and in response to three depleted nitrogen concentrations **(B)** after 2, 6 and 10 days of incubation in response to five different temperature degrees. The error bars indicate mean ± SE (n = 3). The effects of the treatments were tested by one-way analysis of variance (ANOVA). Means were compared between the treatments using the LSD (least significant difference) test at the 0.05 probability level.

**Figure 2:**
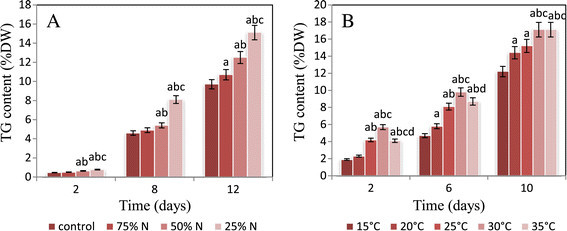
**Triglyceride content of** ***Nannochloropsis salina*** **.** The triglyceride contents were measured **(A)** after 2, 8 and 12 days of cultivation under optimal conditions and three depleted nitrogen concentrations **(B)** after 2, 6 and 10 days of cultivation under five different temperature degrees. The error bars indicate mean ± SE (n = 3). The effects of the treatments were tested by one-way analysis of variance (ANOVA). Means were compared between the treatments using the LSD (least significant difference) test at the 0.05 probability level.

**Figure 3:**
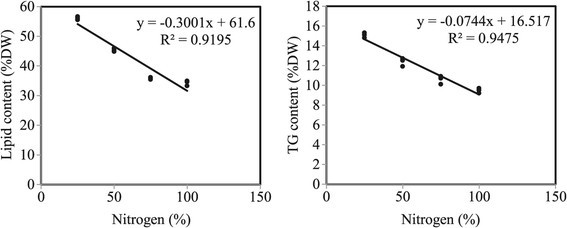
**Statistical data relating to nitrogen limitation.** The correlation between lipid and triglyceride contents as dependent variables and nitrogen concentrations as independent variable was performed using simple linear regression analysis.

Although *N. salina* maintained the ability to grow under nitrogen depletion with a reduced rate in cell number and dry weight comparing to that grown in control (Table 1), nitrogen depletion induced an increase in the lipid content which is correlated with the accumulation of triglycerides with highest percentages after 12 days of culturing (Figures 1 and 2). The maximum lipid and triglyceride contents (56.1and 15.1% of dry weight respectively) were gained at 25% nitrogen concentration followed by 50% nitrogen concentration (45.2 and 12.5 of dry weight respectively), while the minimum contents (36.1 and 10.7% of dry weight respectively) were obtained at 75% nitrogen concentration. With regard to the control, the lipid and triglyceride contents were 34.6 and 9.7% of dry weight respectively. Simple regression analysis (Figure 3) showed significant relationships between lipid and triglyceride contents and nitrogen concentrations giving 91% and 94% (simple regression: y_lipids_ = −0.300x + 61.6, R^2^ = 0.919, and y_triglycerides_ = −0.074x + 16.51, R^2^ = 0.947) of the variation, respectively. Consequently, lipids and triglycerides tend to accumulate more in the cells under condition of nitrogen limitation.

### Effect of variation in temperature

To study the combined effect of nitrogen limitation and variation in temperature on batch growth of *N. salina*, cells grown in 25% nitrogen concentration were re-cultivated under different temperatures (15, 20, 25, 30 and 35°C) up to the stationary phase. The maximum algal growth was achieved on the 10^th^ day at 30°C. Later, the micro-algal growth was gradually dropped and the pale green color of cells was noticed. By comparing the results of growth measurements to the control (Table 1), decrease in cell number, optical density and dry weight of *N. salina* accompanied by increase in the lipid productivity were marked. Number of cells were ranged from 6.9 × 10^6^ to 8.8 × 10^6^ /ml culture with optical density of 0.53 and 0.72 and dry weight of 0.42 and 0.53 respectively. However, the maximum (0.431 g L^−1^ day^−1^) and minimum (0.311 g L^−1^ day^−1^) lipid productivity were acquired at 30°C and 15°C respectively after 10 days of culturing.

**Figure 4:**
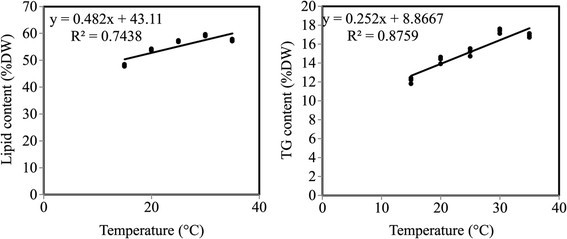
**Statistical data relating to variation in temperature.** The correlation between lipid and triglyceride contents as dependent variables and cultivation temperature degrees as independent variable was carried out via simple linear regression analysis.

Lipid and triglyceride contents on the second, sixth and tenth days of culturing were demonstrated in Figures 1 and 2. Reduction in nitrogen concentration up to 25% accompanied by raising the temperature from 15 to 35°C resulted in an increase in both lipid and triglyceride contents with maximum values at 30°C. Considerable increase in lipid and triglyceride contents was observed since the biomass decreased with respect to the control. The lipid and triglyceride contents were 59.3 and 17.1% of dry weight respectively yielded from a biomass of 0.53 g L^−1^ at 10^th^ day under 25% N-limitation and 30°C, whereas under control condition, they were found to be 34.6 and 9.7% of dry weight respectively with biomass of 0.61 g L^−1^ at 12^th^ day. Simple regression analysis (Figure 4) explained significant relationships between lipid and triglyceride contents and temperature with 74% and 87% (simple regression, y_lipid_ = 0.482x + 43.11, R^2^ = 0.743, y_triglyceride_ = 0.252x + 8.866, R^2^ = 0.875).

## Discussion

The optimization of culture condition for lipid production was affected by many interrelated factors. Significant differences in growth and cellular components of microalgal cells have been observed depending on how the alga will act in response to variations in culture conditions such as limitation or starvation of essential nutrients (Pinto et al. [2003]; Ip and Chen [2005]). Nitrogen is the most critical macronutrient affecting growth and lipid metabolism in algae, since it is an essential constituent of functional processes and cell structure (Sharma et al. [2012]). A general trend of lipid accumulation, mostly triglycerides, in response to nitrogen deficiency has been observed in several species of different microalgae (Hsieh and Wu [2009]; Yeh and Chang [2011]; Sun et al. [2014]). Furthermore, temperature is one of the key factors that affects the growth, lipids and the types of fatty acids produced by microalgae (Renaud et al. [2002]; Converti et al. [2009]; Taoka et al. [2009]). In the present investigation, *N. salina* was characterized in terms of their responses to reduction in nitrogen concentration and different cultivation temperatures. Cell counting, optical density and biomass dry weight are effortless methods easy to use for *N. salina* growth evaluation. The relationship between cell number and optical density is dependent on culture conditions, for instance culture media and cell age. In fact, the size and weight of each cell is not always the same. Results indicated that cell counts, optical density and biomass dry weight of the tested alga decreased when grown under reduced nitrogen concentrations simultaneously with enhancement in lipid productivity, total lipid and triglyceride contents. This result is in accordance with Yeesang and Cheirsilp ([2011]) who reported the loss of biomass when green alga, *Botrycoccus sp.* was exposed to nitrogen deficient conditions. Mandal and Mallick ([2009]) and Gouveia and Oliveira ([2009]) have also reported decreased growth pattern in *Scenedesmus obliquus*, under nitrogen deficient conditions as well as for *Chlorella pyrenoidosa* (Nigam et al. [2011]).

In this study, there is an inverse relationship between lipid content and nitrogen concentration. *N. salina* was able to stay alive under the whole tested nitrogen concentrations and induces increase in cellular lipid content. This is attributable to the unusual pattern of lipids together with the ability to adapt lipid metabolism efficiently in response to changes in cultivation conditions (Guschina and Harwood [2006]). Culture aging or senescence also affects lipid and fatty acid content and composition. The total lipid content of cells increased with age in the green alga *Chlorococcum macrostigma* (Collins and Kalnins [1969]), and the diatom *Thalassiosira fluviatillis* (Conover [1975]) and *Coscinodiscus eccentricus* (Pugh [1971]). However, this increase in total lipids in *N. salina* cells was mainly of neutral lipids, mostly triglycerides and the maximum increase was noticed for the duration of stationary phase. This is in agreement with Bigogno et al. ([2002]) who reported that triglycerides increased in the green alga *Parietochloris incise* from 43% in the logarithmic phase to 77% in the stationary phase. However, this increase in triglycerides may due to the alteration in lipid metabolism from membrane lipid synthesis to storage of neutral lipids. However, biosynthesis and conversion of some existing membrane polar lipids into triglycerols can contribute to the enhancement in triglyceride (Xiao et al. [2013]). Therefore, triglycerides may account for as much as 80% of the total lipid content in the cell (Tornabene et al. [1983]; Suen et al. [1987]; Tonon et al. [2002]; Hu et al. [2008]).

Presented here, data of lipid productivity was contrary associated with tested nitrogen concentrations indicating that mechanisms associated with nitrogen metabolisms might be involved in lipid biosynthesis (Wagenen et al. [2012]) and accordingly enhancement of lipid productivity. Cultivation of *N. salina* under reduced nitrogen concentrations generates the necessity for nitrogen and encourages the accumulation of preserved lipids and increasing in lipid productivity. However, lipid accumulation may be lower in higher nitrogen concentrations and consequently resulting in a lesser amount of lipid productivity. Comparable results showed that transferring algal biomass from a nitrogen-sufficient phase to a nitrogen-deficient phase induces lipid production and lipid productivity (Su et al. [2011]).

In this work, the combined effect of nitrogen-limited conditions (25% of the original concentration in the medium) and different temperature degrees was studied. The microalga exhibits reduction in growth and accretion in lipid productivity when compared to control. Moreover, low temperatures had a minimal effect while high temperatures had a maximal effect on lipids and triglycerides improvement. The ability of *N. salina* to grow under the entire range of the studied temperature degrees and 25% nitrogen concentration illustrates that the combined effect of these two studied stressors create an additional response to accumulate cellular lipids and triglycerides and accordingly increase in lipid productivity. Illman et al. ([2000]) and Liu et al. ([2008]) have revealed that the quantity and quality of lipids within the cell may well differ in relation to changes in growth conditions such as temperature or light intensity. Actually, temperature affects the physiological processes by changing the rate of chemical reactions and the stability of cellular components (Sandnes et al. [2005]; Wagenen et al. [2012]). However, lipid production and lipid productivity is a strain-specific function of physiological responses to many factors such as cultivation temperature (Griffiths and Harrison [2009]; Wagenen et al. [2012]). The lipid content in the chrysophytan *Ochromonas danica* (Aaronson [1973]) and the eustigmatophyte *N. salina* (Boussiba et al. [1987]) increases with increasing temperature. In contrast, no significant change in the lipid content was observed in *Chlorella sorokiniana* grown at various temperatures (Patterson [1970]). In this work, simple regression analysis is used to assess the relative impact of nitrogen concentrations and temperatures as variables on lipid and triglyceride production in *N. salina*. Referring to statistical results, reduction of nitrogen concentration to 75% of the original medium significantly explained the increase in lipid and triglyceride produced, while less significant was observed when testing the combined effect between 25% nitrogen concentration and temperature of 30°C. This may be attributed to the lipid metabolism, particularly the biosynthetic pathways of lipid and triglycerides. Synthesis and accumulation of lipid correlated with significant changes in triglycerides in the cell occur when algae are cultivated under stress conditions. This accumulation forced by chemical or physical stimuli, either individually or in combination (Hu et al. [2008]). Based on this study, nitrogen limitation needs to be more considered rather than combination with temperature when monitoring lipids and triglycerides production in *N. salina*.

## Conclusion

Of particular interest was the influence of nitrogen depletion and variation of temperature on lipids as well as triglycerides production in *N. salina*. As nitrogen concentration in the medium was reduced by 75% and at temperature of 30°C, an increase in lipid and triglyceride contents with high lipid productivity was noticed. Based on dry weight, algal biomass can serve as lipid-rich feedstock with increased lipid content (59.3%) compared to control (34.6%). Simple regression provides significant differences in percentages between variables. For application at commercial scale, future work should be directed to additional practice to improve algal lipid yield per unit time.

## Authors’ original submitted files for images

Below are the links to the authors’ original submitted files for images. Authors’ original file for figure 1 Authors’ original file for figure 2 Authors’ original file for figure 3 Authors’ original file for figure 4
