# Supplementary figures and images for: Lipid accumulation in response to nitrogen limitation and variation of temperature in Nannochloropsis salina
Source: Bot Stud. 2015 Apr 8;56:6. doi: 10.1186/s40529-015-0085-7 (PMC5432932; doi:10.1186/s40529-015-0085-7)

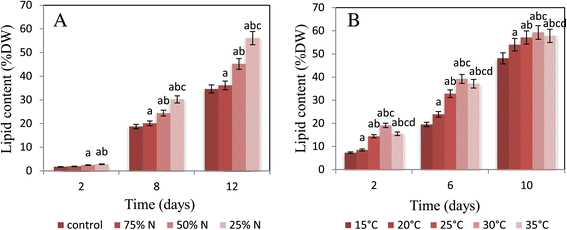

Supplement: Supplementary file 1 — Authors’ original file for figure 1 [file 40529_2015_85_MOESM1_ESM.gif]

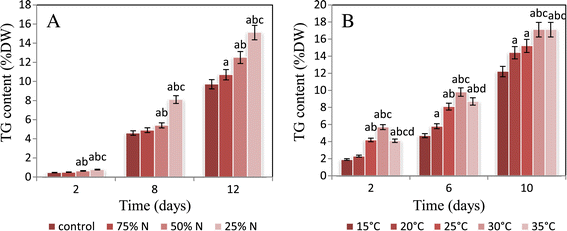

Supplement: Supplementary file 2 — Authors’ original file for figure 2 [file 40529_2015_85_MOESM2_ESM.gif]

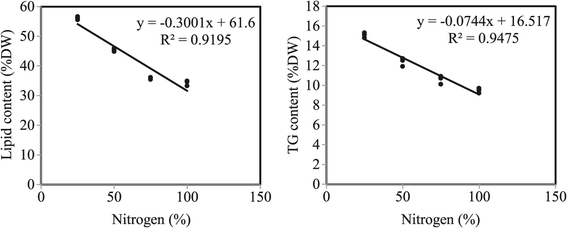

Supplement: Supplementary file 3 — Authors’ original file for figure 3 [file 40529_2015_85_MOESM3_ESM.gif]

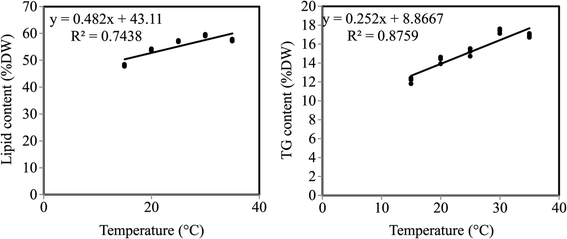

Supplement: Supplementary file 4 — Authors’ original file for figure 4 [file 40529_2015_85_MOESM4_ESM.gif]
